# Thiostrepton induces ferroptosis in pancreatic cancer cells through STAT3/GPX4 signalling

**DOI:** 10.1038/s41419-022-05082-3

**Published:** 2022-07-20

**Authors:** Weifan Zhang, Mengyuan Gong, Wunai Zhang, Jiantao Mo, Simei Zhang, Zeen Zhu, Xueni Wang, Bo Zhang, Weikun Qian, Zheng Wu, Qingyong Ma, Zheng Wang

**Affiliations:** 1 Department of Hepatobiliary Surgery, The First Affiliated Hospital of Xi’an Jiaotong University, Xi’an, Shaanxi 710061 China; 2 Pancreas Center, Xi’an Jiaotong University, Xi’an, Shaanxi 710061 China

**Keywords:** Cancer prevention, Cancer therapy

## Abstract

Ferroptosis is a new form of regulated cell death that is mediated by intracellular iron and ester oxygenase, and glutathione-dependent lipid hydroperoxidase glutathione peroxidase 4 (GPX4) prevents ferroptosis by converting lipid hydroperoxides into nontoxic lipid alcohols. Although thiostrepton (TST) has been reported to exert antitumor effects, its role in pancreatic cancer and the underlying mechanisms remain unclear. In this study, we found that TST reduced the viability and clonogenesis of pancreatic cancer cell lines, along with intracellular iron overload, increasing reactive oxygen species (ROS) accumulation, malondialdehyde (MDA) overexpression, and glutathione peroxidase (GSH-PX) depletion. Mechanistically, chromatin immunoprecipitation (ChIP) and dual luciferase reporter gene assays were used to confirm that signal transducer and activator of transcription 3 (STAT3) binds to the GPX4 promoter region and promotes its transcription, whereas TST blocked GPX4 expression by regulating STAT3. Finally, in vivo experiments revealed that TST inhibited the growth of subcutaneously transplanted tumours and had considerable biosafety. In conclusion, our study identified the mechanism by which TST-induced ferroptosis in pancreatic cancer cells through STAT3/GPX4 signalling.

## Introduction

Pancreatic cancer is a malignant tumour with an insidious onset and rapid progression that prevents most patients with pancreatic cancer from undergoing surgery. Pancreatic ductal adenocarcinoma (PDAC) is the most common pathological type of pancreatic cancer, accounting for ~90% of cases. Moreover, patients with PDAC are not sensitive to chemotherapy, immunotherapy, and other treatments, making the treatment approach relatively deficient [1, 2]. Hence, an understanding of the biological mechanisms and molecular mechanisms is urgently needed to develop new therapeutic strategies for the comprehensive treatment of pancreatic cancer.

Ferroptosis is a new form of cell death that is iron-dependent and characterized by lipid peroxidation [3]. A number of factors directly or indirectly promote ferroptosis; biochemically, intracellular iron overload, intracellular glutathione (GSH) depletion, decreased activity of glutathione peroxidase (GPX), and accumulation of lipid reactive oxygen species (ROS) contribute to ferroptosis, and lipid peroxides are not metabolized in reduction reactions, promoting oxidative cell death. Microstructurally, ferroptosis is often associated with a reduced mitochondrial volume, increased bilayer membrane density and the reduction or disappearance of mitochondrial cristae. At the genetic level, ferroptosis is regulated by a variety of pathways. Glutathione peroxidase 4 (GPX4) is a GPX family member that transforms lipid peroxides to lipid alcohols using glutathione (GSH) as a cofactor, thereby suppressing ferroptosis [4]. Cysteine is the rate-limiting precursor for GSH synthesis, and SLC7A11, a multipass transmembrane protein, mediates cystine/glutamate antiporter activity in the system and regulates GSH synthesis to affect ferroptosis [5]. Therefore, some small molecules targeting these pathways were shown to trigger and restrain ferroptosis. For example, RAS-selective lethal 3 (RSL3) induces ferroptosis by inhibiting GPX4, while ferrostatin-1 (Fer-1) is a synthetic antioxidant that inhibits cell death through reducing mechanisms to prevent membrane lipid damage.

The role of ferroptosis in pancreatic cancer has been gradually reported with the development of new analytical methods. Studies have shown that endoplasmic reticulum (ER)-associated molecular chaperone, HSPA5, binds to the GPX4 protein and prevents its degradation to inhibit ferroptosis. Moreover, HSPA5 knockdown also inhibits artesunate-induced upregulation of the GPX4 protein, thereby rescuing the sensitivity of PDAC cells to artesunate-induced ferroptosis [6]. KRAS and TP53 mutations are common in pancreatic cancer, and mutations of these genes are also associated with ferroptosis. Therefore, a study of the regulatory mechanism of ferroptosis may provide a potential breakthrough in the treatment of pancreatic cancer [7, 8].

Thiostrepton (TST) is a protein translation inhibitor that is essentially active against Gram-positive bacteria and some Gram-negative bacteria [9]. In previous research, TST has been reported to inhibit FOXM1 and the malignant behaviour of a variety of tumours, including colorectal cancer, breast cancer, lung cancer, acute lymphoblastic leukaemia, ovarian cancer, and even pancreatic cancer [10–15]. However, how it affects pancreatic cancer and the underlying mechanisms remain unclear. Signal transducer and activator of transcription 3 (STAT3) mediates the expression of a variety of genes in response to cell stimuli and thus plays a key role in many cellular processes, such as cell growth and apoptosis. Zhong et al. reported that Elabela alleviates ferroptosis by modulating IL-6/STAT3/GPX4 signalling [16]. Other studies have also confirmed that STAT3 regulates ferroptosis in various forms, but its role in pancreatic cancer has not been reported.

In this study, we investigated the effect of TST on ferroptosis in pancreatic cancer. We found that TST reduced the viability and clonogenesis of pancreatic cancer cells and induced intracellular iron overload, ROS accumulation, MDA overexpression, and glutathione peroxidase (GSH-PX) depletion. Mechanistically, STAT3 bound to the GPX4 promoter region to promote its transcription, and TST induced ferroptosis by inhibiting STAT3 protein expression. Moreover, TST promoted ferroptosis and had certain biosafety in vivo. Since ferroptosis exhibited potential clinical application value, our findings might provide new strategies for the comprehensive treatment of pancreatic cancer.

## Materials and methods

### Cell culture and treatment

The pancreatic cancer cell lines, Panc-1, BxPC-3, MIA PaCa-2, and the human pancreatic ductal epithelial cell line hTERT-HPNE were purchased from the Chinese Academy of Sciences Cell Bank of Type Culture Collection (CBTCCCAS, Shanghai, China). Panc-1 and MIA PaCa-2 cells were cultured in Dulbecco’s modified Eagle’s medium (DMEM; Gibco; Thermo Fisher Scientific, USA), and BxPC-3 cells were cultured in RPMI-1640 supplemented with 10% foetal bovine serum (FBS) at 37 °C with 5% CO_2_. Thiostrepton (TST, purity ~99%), RSL3 (purity ~99%), and ferrostatin-1 (Fer-1, purity ~99%) were purchased from MedChemExpress (MCE, USA) and dissolved in dimethyl sulfoxide (DMSO). hTERT-HPNE cells were cultured in 75% DMEM supplemented with 5% foetal bovine serum, 10 ng/ml human recombinant EGF, 5.5 mM d-glucose (1 g/L), and 750 ng/ml puromycin. Cells were treated with various concentrations of TST in 1% FBS for 24 or 48 h at 37 °C with 5% CO_2_.

### Cell proliferation and colony formation assays

For assessments of cell viability, hTERT-HPNE, Panc-1, MIA PaCa-2, and BxPC-3 cells were seeded in 96-well plates at a density of 3000 cells per well and treated with various concentrations of TST (0–250 μM) for 48 h. Cells were treated with RSL3 (3 μM) or Fer-1 (5 μM) for 24, 48 and 72 h to assess cell proliferation. Next, the cells were treated with 10 μL of CCK-8 reagent for 2 h and assessed by measuring the absorbance at 450 nm using a microplate reader (Molecular Device, Sunnyvale, CA, USA). Pancreatic cancer cells (Panc-1, MIAPaCa-2, and BxPC-3) were seeded onto 6 cm dishes at a density of 2000 cells per dish, cultured for 48 h, and treated with different concentrations of TST, RSL3, or Fer-1 for 2 weeks. Then, the cells were washed with phosphate-buffered saline (PBS), fixed with 4% paraformaldehyde and stained with a crystal violet solution for 10 min. The number of colonies was counted in five random fields under a microscope.

### Intracellular iron and reactive oxygen species (ROS) and assays

For the intracellular iron assay, pancreatic cancer cells were seeded in 24-well plates and treated with various concentrations of TST, and then the Mito-FerroGreen kit (DOJINDO, Kyushu, Japan) was applied to detect the intracellular iron level of cells. The specific steps are carried out in accordance with the instructions. For ROS assay, Panc-1, MIA PaCa-2, and BxPC-3 cells were seeded in 24-well plates and treated with various concentrations of TST, RSL3, or Fer-1 for 24 h; then, the fluorescent probe DCFH-DA provided in the ROS Assay Kit (Beyotime, Shanghai, China) was added to the cells. Next, the cells were incubated for 20 min in the cell culture incubator at 37 °C and washed with serum-free cell culture medium three times, and ROS levels were observed with a laser scanning confocal microscope (Nikon A1R/A1).

### Lipid peroxidation malondialdehyde(MDA) Assay and Total Glutathione Peroxidase Assay

Panc-1, MIA PaCa-2 and BxPC-3 cells were seeded in 10 cm dishes and treated with various concentrations of TST, RSL3 or Fer-1 for 24 h. The cells were then collected, homogenized, lysed, and centrifuged at 10,000–12,000×*g* for 10 min to obtain the supernatant. The Lipid Peroxidation MDA Assay Kit (Beyotime, Shanghai, China) was used to detect the MDA content in cells. In cell lysates, MDA reacts with thiobarbituric acid (TBA) to generate MDA–TBA adducts. Then, the absorbance was measured at 532 nm with a microplate reader, and the concentration of MDA was determined by comparing the value to a standard curve. According to instructions of the Total Glutathione Peroxidase Assay kit (Beyotime, Shanghai, China), cell lysates were added to the 96-well plate, and then detection buffer and GSH-PX detection working solution were added. After incubation at room temperature for 15 min, the peroxide reagent solution was added, and samples were measured using a spectrophotometer to determine A340 immediately after mixing. At this time, the initial value was recorded. After incubation for 10 min, the value was measured again. Finally, the glutathione peroxidase activity in the samples was calculated according to the formula provided in the manual.

### Immunofluorescence (IF) staining

Panc-1, MIA PaCa-2 and BxPC-3 cells were cultivated in 24-well plates after treatment, fixed with 4% paraformaldehyde for 10 min, washed with PBS three times, treated with permeabilization solution (1% Triton X-100 in PBS), washed with PBS again, and blocked with 5% bovine serum albumin (Sigma-Aldrich, Germany) for 1 h. The primary antibody was added to the 24-well plate and mixed overnight at 4 °C. Next, the samples were washed with PBS three times, incubated with a fluorescein isothiocyanate-conjugated AffiniPure goat anti-rabbit IgG secondary antibody (dilution, 1:200; Beijing Zhongshan Golden Bridge Biotechnology, China) for 60 min, and then stained with DAPI (1:10,000) for 10 min in the dark. A laser scanning confocal microscope (Nikon A1R/A1) was used to observe the samples.

### RNA isolation and relative quantitative real-time PCR (qRT–PCR)

Total RNA was extracted from different cells with a Fastgen2000 RNA isolation system (Fastgen, Shanghai, China) according to the manufacturer’s protocol. Complementary DNA was synthesized using reverse transcription kits (TaKaRa). Quantitative real-time RT–PCR (qRT–PCR) was performed with a Bio-Rad iQTM5 system (Bio-Rad). The 2^−ΔΔCt^ method was used to analyse qRT–PCR data. The information of primers was listed below: GPX4-F: CAGTGAGGCAAGACCGAAGT; GPX4-R: GGGGCAGGTCCTTCTCTATC.

### Western Blotting assay

The PDAC cell lines Panc-1, MIA PaCa-2, and BxPC-3 were seeded in 6 cm dishes, cultured for 24 h, and then treated with various concentrations of TST for 48 h. Cells and tumour tissues were lysed with RIPA buffer. SDS–PAGE gels were used to electrophorese the total protein samples, which were then transferred to PVDF membranes (Roche, Penzberg, Germany). The membranes were blocked with QuickBlock™ Blocking Buffer for 20 min and incubated overnight at 4 °C with the primary antibody. After three washes with PBST, the membranes were incubated with a secondary antibody for 1 h at room temperature. Finally, the membranes were washed with PBST three times, and a ChemiDoc XRS System (Bio-Rad, CA, USA) was used to detect the expression of proteins with an enhanced chemiluminescence (ECL) kit (NCM Biotech, Suzhou, China). The information of primary antibodies was listing as below: anti-GPX4 (Abcam, CAT# ab125066); anti-STAT3 (Abcam, CAT# ab68153); anti-pSTAT3 (Zenbio, CAT#381552), anti-SLC711A (Proteintech, CAT#26864-1-AP); anti-FOXM1 (Santa Cruz, CAT# sc-376471); and anti-β-Actin (Proteintech, CAT# 66009-1-Ig).

### Chromatin immunoprecipitation (ChIP) and dual luciferase reporter assay

ChIP was performed using the SimpleChIP Enzymatic Chromatin IP kit (Cell Signaling Technology). HEK293T cells were seeded in 10 cm dishes, cross-linked with the reagent when they grew to 90% confluence, and lysed with SDS buffer followed by ultrasonication. Then, ultrasound was used to break the DNA into fragments of 100–500 bp, and specific antibodies or normal mouse IgG were used to pull down the DNA. After washing with high salt and low salt buffers, DNA was eluted and decrosslinked, and enriched sequences were examined using qPCR. And GPX4 primers used in chip assay were shown in Supplemental Table 1. For the promoter luciferase reporter assay, the promoter region containing only WT1 and the promoter region containing only MUT1 were synthesized and cloned into the pmirGLO basic luciferase reporter (Miaolingbio, Wuhan, China). Luciferase activity was measured with the Dual Luciferase Assay System (Beyotime, Shanghai, China). Renilla luciferase activity was normalized to firefly luciferase activity.

### Subcutaneous tumour model

The MIA PaCa-2 cell line was used to build the subcutaneous tumour model. MIA PaCa-2 cells were resuspended in a serum-free medium at a density of 2 × 10^7^ cells/mL and mixed with matrix at a ratio of 1:1. Next, 100 μL of the cell suspension was injected subcutaneously into female BALB/c nude mice for 4 weeks age. When the tumours were macroscopic (approximately one week), the mice were divided into three groups (five mice per group) according to the random table method (Number 1–15): the control group, TST group, and TST + Fer-1 group (three times a week). The size of the tumour was measured three times a week. After 3 weeks of drug administration, blood was collected from the mouse eyeballs, and the serum was used to measure alanine transaminase (ALT), aspartate aminotransferase (AST), creatinine (CRE), and urea nitrogen (BUN) levels to evaluate the safety of the drug. The subcutaneous tumours were removed, weighed, fixed, and proteins extracted. The heart, liver, spleen, lung and kidney were removed and immobilized, and HE staining was performed to evaluate the potential toxicity of the drug to the organ. All protocols were approved by the Ethics Committee of the First Affiliated Hospital of Xi’an Jiaotong University, Xi’an, China.

### Statistical analysis

All statistical analyses were performed using GraphPad Prism version 8.0 software (GraphPad Software, USA). All data are reported as the means ± SD of triplicate experiments, and the differences between the two groups were compared using the two-tailed Student’s *t*-test. Comparisons between multiple groups were performed using one-way ANOVA, and *P* < 0.05 was considered statistically significant.

## Results

### Thiostrepton reduces the viability and clonogenesis of pancreatic cancer cells

**Fig. 1:**
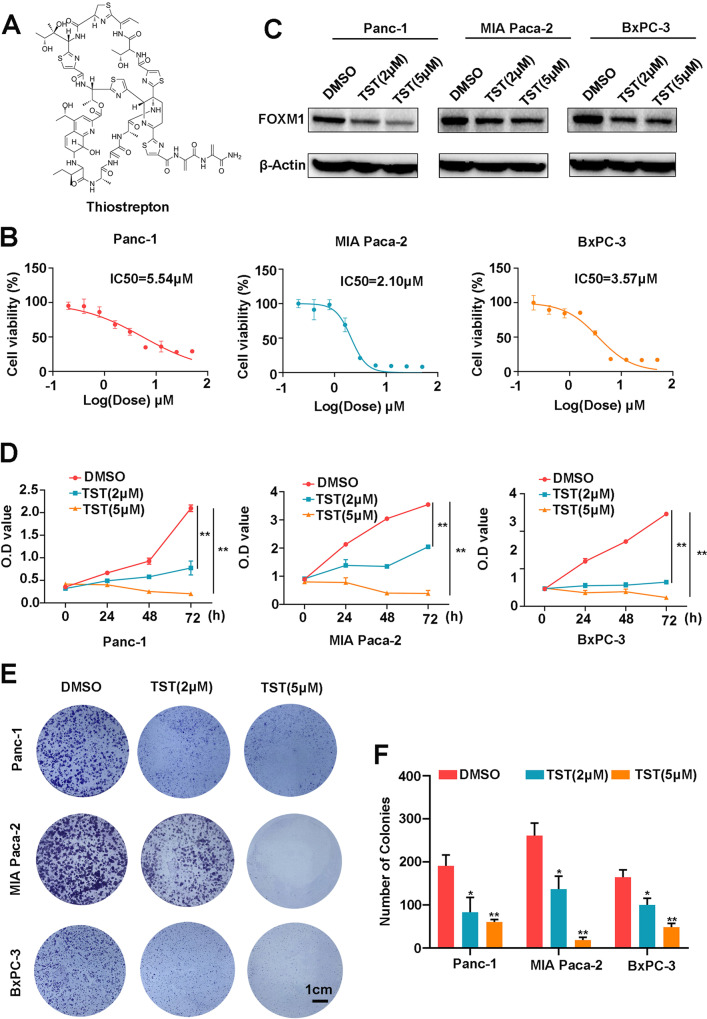
Thiostrepton reduces the viability and clonogenesis of pancreatic cancer cells. **A** Chemical structure of TST. **B** Panc-1, MIA PaCa-2, and BxPC-3 cells were treated with TST (0–200 μM) for 24 h, and cell viability was determined using the CCK-8 assay. **C** Western blotting analysis detected the level of the FOXM1 protein in different treatment groups (DMSO, 2 or 5 μM). **D** OD value of the CCK-8 assay conducted at 0, 24, 48, and 72 h using pancreatic cancer cells treated with different concentrations of TST (DMSO, 2, or 5 μM). **E** and **F** Panc-1, MIA PaCa-2, and BxPC-3 cells were treated with different doses of TST (DMSO, 2 or 5 μM), and cell clonogenesis was measured by using a colony formation assay (*P < 0.05 and **P < 0.01).

TST, known as thiopeptide, possesses anticancer properties, and its chemical structure is shown in Fig. 1A. Pancreatic cancer cells have a strong ability to proliferate; therefore, we first evaluated the inhibitory effect of TST on cell proliferation. Using the CCK-8 assay, we measured the viability of Panc-1, MIAPaCa-2 and BxPC-3 pancreatic cancer cells treated with different doses of TST. TST significantly reduced the growth of pancreatic cancer cells in a dose-dependent manner, and the half-maximal inhibitory concentrations (IC_50_s) in Panc-1, MIA PaCa-2, and BxPC-3 cells were 5.54, 2.10, and 3.57 μM, respectively (Fig. 1B). In addition, TST has been indicated to be a selective inhibitor of FOXM1; therefore, we used two concentrations of TST (2 and 5 μM) to treat pancreatic cancer cells. Western blotting results showed that the two drug concentrations we selected significantly reduced factor forkhead box M1 (FOXM1) protein expression levels (Fig. 1C). Then, the cells were further treated with the proven effective dose of TST, and the CCK-8 was performed at the 0, 24, 48, and 72 h; the OD values showed that TST prevented pancreatic cancer cells from growing in a time-dependent manner (Fig. 1D). Finally, using a colony formation assay, we found that TST significantly inhibited the clonogenesis of Panc-1, MIAPaCa-2 and BxPC-3 cells (Fig. 1E, F). All these findings suggested that TST suppressed pancreatic cancer cell proliferation.

### Thiostrepton promotes ferroptosis in pancreatic cancer cells

**Fig. 2:**
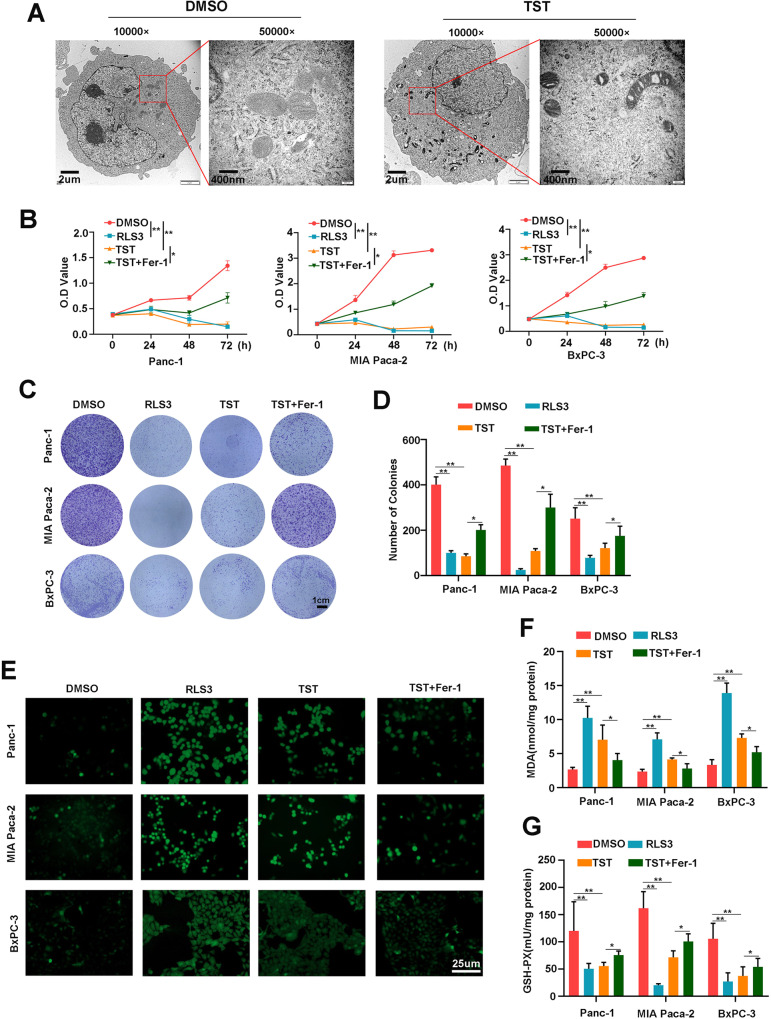
Thiostrepton promotes ferroptosis in pancreatic cancer cells. **A** TEM imaging was conducted to control pancreatic cancer cells and TST (2 µM) treated pancreatic cancer cells. **B** OD value of the CCK-8 assay was conducted at 0, 24, 48, and 72 h using Panc-1, MIA PaCa-2, and BxPC-3 cells from different groups (DMSO, RSL3, TST, and TST + Fer-1). **C** and **D** The clonogenesis of pancreatic cancer cells in different treatment groups was measured using the colony formation assay. **E** ROS levels were detected in pancreatic cancer cells from different treatment groups with a ROS Assay Kit. **F** and **G** MDA and GSH-PX levels were detected in the different groups of treated Panc-1, MIA PaCa-2, and BxPC-3 cells. (*P < 0.05 and **P < 0.01).

We fixed pancreatic cancer cell masses after the intervention to explore the mechanism by which TST inhibits the proliferation of pancreatic cancer cells. Transmission electron microscopy was then used to observe the microstructure of the cells. As shown in Fig. 2A, the treated cells presented smaller mitochondria, a higher density of the mitochondrial membrane, and reduced mitochondrial cristae. Moreover, to confirm whether intracellular iron levels could further reflect the existence of ferroptosis, we measured the intracellular iron levels after TST treatment, and the results showed that intracellular iron levels in the treated group increased significantly (Fig. S1). Therefore, we hypothesized that the mechanism by which TST inhibits cell proliferation includes ferroptosis. The ferroptosis inducer RSL3 and the inhibitor Fer-1 were used as experimental interventions, and we divided the cells into four groups (DMSO, RSL3, TST, and TST + Fer-1) and observed the proliferation of cells in each group to further confirm this hypothesis. RSL3 and TST both reduced the proliferation of Panc-1, MIAPaCa-2 and BxPC-3 cells, while Fer-1 significantly rescued proliferation (Fig. 2B). Then, the colony formation assay was performed on cells from the four groups. We further confirmed that RSL3 and TST significantly inhibited the clonogenesis of pancreatic cancer cells and that Fer-1 rescued this inhibition (Fig. 2C, D). ROS accumulation is regarded as one hallmark of ferroptosis, and we further speculated that the TST intervention might promote ROS production. The experiment was also performed on cells from the four groups, and 24 h after the intervention, we measured intracellular ROS levels using the oxidation-sensitive fluorescent probe DCFH-DA. Our results showed that RSL3 and TST significantly increased ROS production, and Fer-1 also reversed this change (Fig. 2E). MDA and GSH-PX play key roles in maintaining the balance of oxidation and reduction, and both exhibit corresponding changes when ferroptosis occurs. We then measured MDA levels in different intervention groups, and MDA levels increased significantly in the RSL3 and TST groups but were reduced by Fer-1 (Fig. 2F). Finally, GSH-PX levels were measured in the three cell lines, and we also confirmed that RSL3 and TST reduced GSH-PX levels, while Fer-1 rescued this change (Fig. 2G). Together, these data supported the hypothesis that TST promotes ferroptosis in pancreatic cancer cells, similar to RSL3.

### Thiostrepton activates the STAT3–GPX4 signalling pathway

**Fig. 3:**
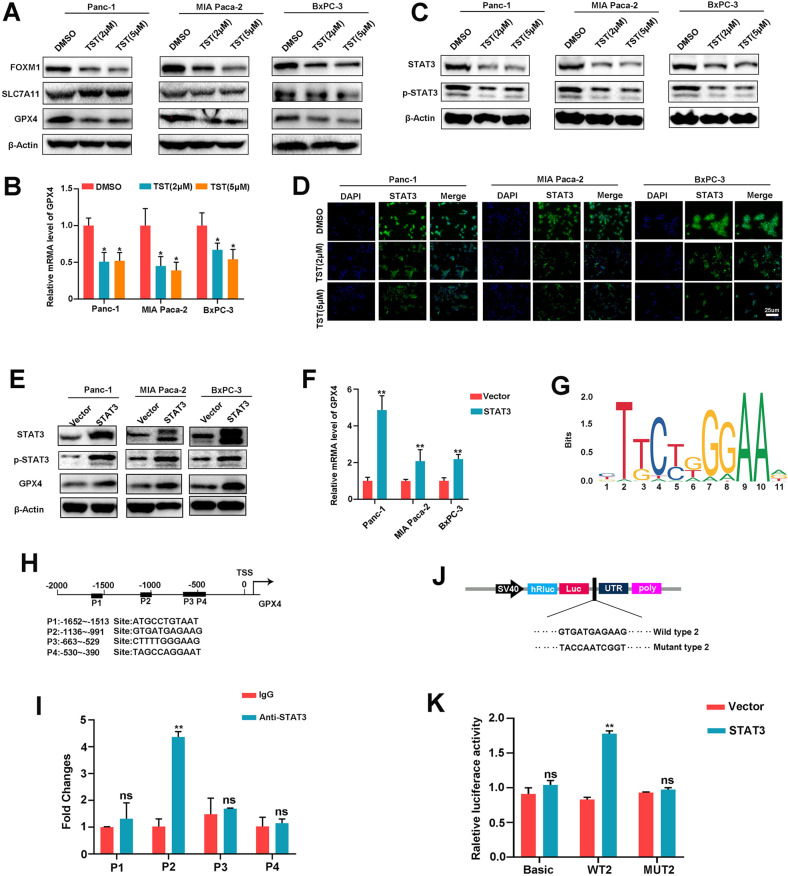
Thiostrepton activates the STAT3–GPX4 signalling pathway. **A** Western blotting analysis of FOXM1, SLC711A, and GPX4 protein levels, **B** qRT-PCR analysis of the GPX4 mRNA expression level, and **C** Western blotting analysis of the STAT3 and p-STAT3 protein levels in Panc-1, MIA PaCa-2, BxPC-3 cells treated with TST (DMSO, 2, or 5 μM). **D** Representative IF images showing the expression of STAT3. The second antibody was labelled with FITC (green), and the nuclei were stained with DAPI (blue). **E** and **F** Western blotting and qRT-PCR analyses of the change in GPX4 expression caused by STAT3 overexpression in pancreatic cancer. **G** and **H** A conserved STAT3-binding motif was predicted by JASPAR, and schematic images of the potential STAT3 binding sites in the promoter of GPX4 are shown. **I** ChIP analysis of STAT3 occupancy at the GPX4 promoter in Panc-1 cells. **J** Schematic images of luciferase reporter plasmids containing STAT3 binding sites (WT and MUT) in the GPX4 promoter region. **K** Luciferase reporter assays of 293T cells overexpressing STAT3 and transfected with reporter plasmids containing WT and MUT GPX4 promoters (*P < 0.05, **P < 0.01, and ns: not significant).

Next, we sought to decipher the molecular mechanisms that may be responsible for the activation of ferroptosis by TST. The cystine/glutamate antiporter SLC7A11 functions to import cysteine for glutathione biosynthesis and antioxidant defences. Glutathione peroxidase 4 (GPX4) attenuates lipid peroxide toxicity and maintains lipid bilayer homoeostasis through its catalytic activity, both of which play important roles in ferroptosis. Therefore, we treated Panc-1, MIAPaCa-2 and BxPC-3 cells, and western blotting experiments showed that SLC7A11 protein levels did not change significantly after the TST intervention, but GPX4 protein levels were significantly downregulated (Fig. 3A). We also verified the GPX4 mRNA level and showed that it was reduced by TST (Fig. 3B). Previous studies have reported that GPX4 is regulated by various molecules, including STAT3. Therefore, we further hypothesized that TST affects GPX4 activity by regulating STAT3. We performed western blotting experiments and confirmed that TST significantly inhibited STAT3 protein expression, and the phosphorylation of STAT3 also decreased accordingly (Fig. 3C). Moreover, based on the results of immunofluorescence staining, TST reduced the expression of STAT3 as well as p-STAT3 both in nucleus and cytoplasm (Figs. 3D, S2). These results suggested that TST may regulate the expression of the GPX4 protein and mRNA through STAT3. Next, we explored how STAT3 modulates GPX4 expression. We overexpressed STAT3 in Panc-1, MIAPaCa-2 and BxPC-3 cells and confirmed that STAT3-upregulated GPX4 mRNA and protein levels (Fig. 3E, F). In addition, we analysed how STAT3 transcriptionally regulates GPX4. The prediction of STAT3 binding sites in the GPX4 promoter using the JASPAR website (http://jaspar.genereg.net/) revealed four putative motifs (P1–P4) in the GPX4 promoter (Fig. 3G, H). Therefore, ChIP–qPCR was designed to confirm the STAT3-binding site in the GPX4 promoter region, and the results confirmed that the P2 site of the promoter region might be the binding site for STAT3 rather than P1, P3, or P4. (Fig. 3I). Finally, we designed luciferase reporter gene plasmids containing the P2 (WT2) site and mutant (MUT2) site (Fig. 3J) and transfected them into 293T cells. By comparing fluorescence values, we found that STAT3 overexpression increased WT2 GPX4 promoter reporter activity but not MUT2 activity (Fig. 3K). Collectively, these data suggest that TST alters GPX4 transcription by decreasing STAT3 expression.

### Thiostrepton-induced ferroptosis depends on the STAT3–GPX4 signalling pathway

**Fig. 4:**
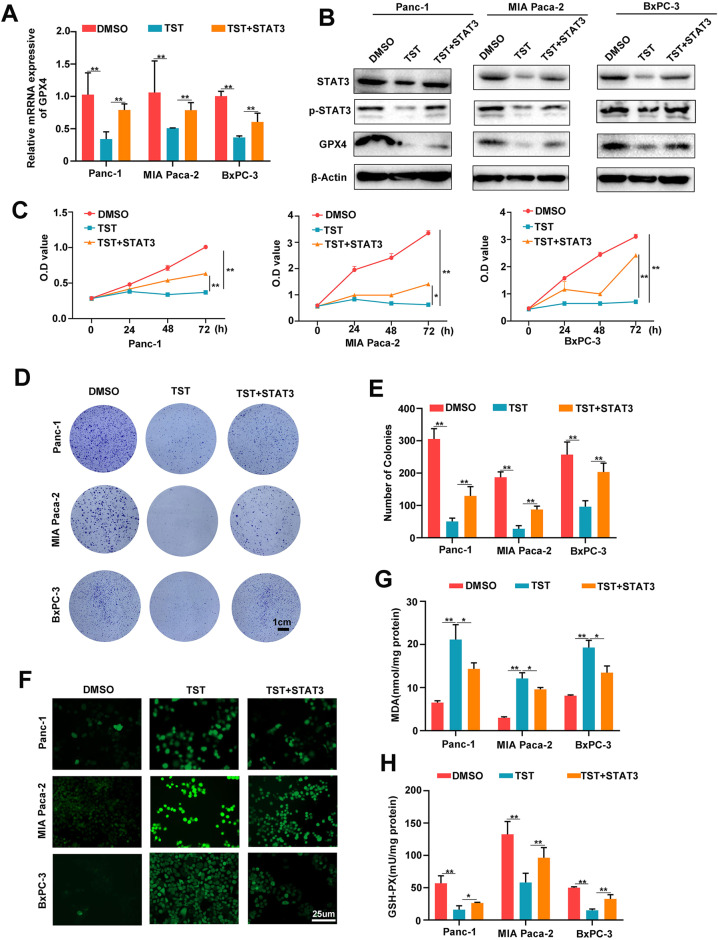
Thiostrepton-induced ferroptosis depends on the STAT3–GPX4 signalling pathway. **A** qRT–PCR analysis of GPX4 mRNA expression. **B** Western blotting analysis of STAT3, p-STAT3 and GPX4 levels in Panc-1, MIA PaCa-2, and BxPC-3 cells receiving different treatments (DMSO, TST, and TST + STAT3). **C** OD value of the CCK-8 assay was conducted at 0, 24, 48, and 72 h using pancreatic cancer cells from different treatment groups. **D** and **E** Clone formation ability and number of pancreatic cancer cells receiving different treatments. **F**, **G**, **H** ROS, MDA and GSH-PX levels were detected in Panc-1, MIA PaCa-2, and BxPC-3 cells from the different treatment groups (*P < 0.05 and **P < 0.01).

Given that TST-induced ferroptosis activated the STAT3–GPX4 signalling pathway in pancreatic cancer cells, we assumed that TST promotes ferroptosis by regulating STAT3-GPX4 signalling. We overexpressed STAT3 in Panc-1, MIAPaCa-2 and BxPC-3 cells and then treated the cells with TST to further explore this hypothesis. An analysis of GPX4 protein and mRNA levels showed that the TST-induced reduction in GPX4 expression was partially rescued by STAT3 overexpression (Fig. 4A, B). The same intervention was then used to observe the proliferation of the three cell lines, and STAT3 overexpression also partially reversed the effect of TST on killing pancreatic cancer cells (Fig. 4C). Similarly, STAT3 overexpression partially rescued the TST-induced decrease in pancreatic cancer cell clonogenesis (Fig. 4D, E). Subsequently, changes in the levels of ferroptosis-related indicators were further examined, and TST increased ROS expression, but this effect was inhibited by STAT3 overexpression (Fig. 4F). Meanwhile, the abnormal levels of MDA and GSH-PX caused by TST were also reversed by STAT3 overexpression (Fig. 4G, H). Taken together, TST induces ferroptosis via the STAT3–GPX4 signalling pathway.

### Thiostrepton benefits to treating pancreatic cancer in vivo

**Fig. 5:**
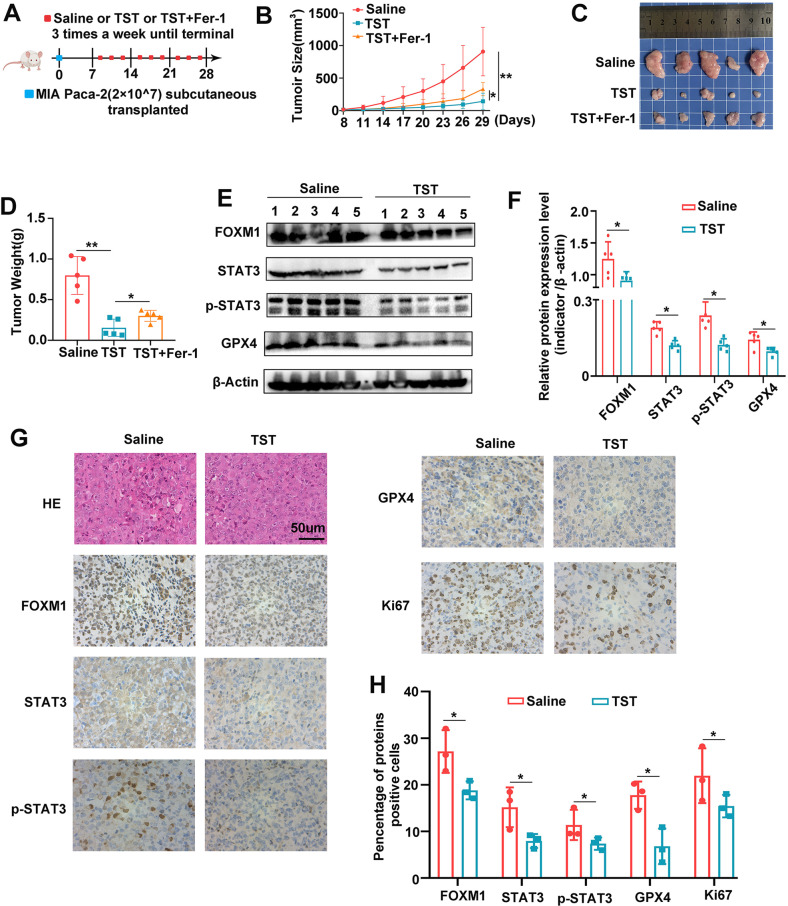
Thiostrepton benefits to treating pancreatic cancer in vivo. **A** Once the tumours reached 2–3 mm in diameter, the subcutaneous xenograft model mice received different treatments (saline, 17 mg/kg TST, TST + 20 mg/kg Fer-1) three times a week until the experiment was terminated. **B** The changes in tumour volume were monitored and are shown for animals receiving different treatments. **C** Representative images of the subcutaneous xenograft model mice. **D** The average weights of the tumours after different treatments. **E** and **F** Western blotting analysis of FOXM1, STAT3, p-STA3 and GPX4 protein levels in tumours from different treatment groups. **G** and **H** The level of FOXM1, STAT3, p-STA3, GPX4, and Ki67 proteins in the indicated xenograft tumours by IHC assay (*P < 0.05 and **P < 0.01).

Subsequently, we investigated the mechanism by which TST induces ferroptosis through the regulation of the STAT3–GPX4-signalling pathway in vivo. MIAPaCa-2 cells transfected with different constructs were subcutaneously injected into BALB/c nude mice. Once the mice developed palpable tumours, they were intraperitoneally injected with TST (17 mg/kg), TST + Fer-1 (5 mg/kg) or the same volume of saline three times a week for 3 weeks (Fig. 5A). TST inhibited tumour growth, and Fer-1, a ferroptosis inhibitor, reversed this effect (Fig. 5B–D). Then, we extracted the proteins from subcutaneously transplanted tumours and conducted western blotting experiments, which also confirmed reduced FOXM1, STAT3, p-STAT3 and GPX4 expression in subcutaneous tumours from the TST treatment group (Fig. 5E, F). To quantitatively assess the index in xenograft tumours, tumour sections were stained for protein expression. As demonstrated in the results, the TST group showed lower FOXM1, STAT3, p-STAT3, GPX4 and Ki67 staining as compared to the control (Fig. 5G, H). Hence, the above data demonstrated the efficacy of the TST for pancreatic cancer in vivo.

### The potential biosafety value of thiostrepton on tumour treatment

**Fig. 6:**
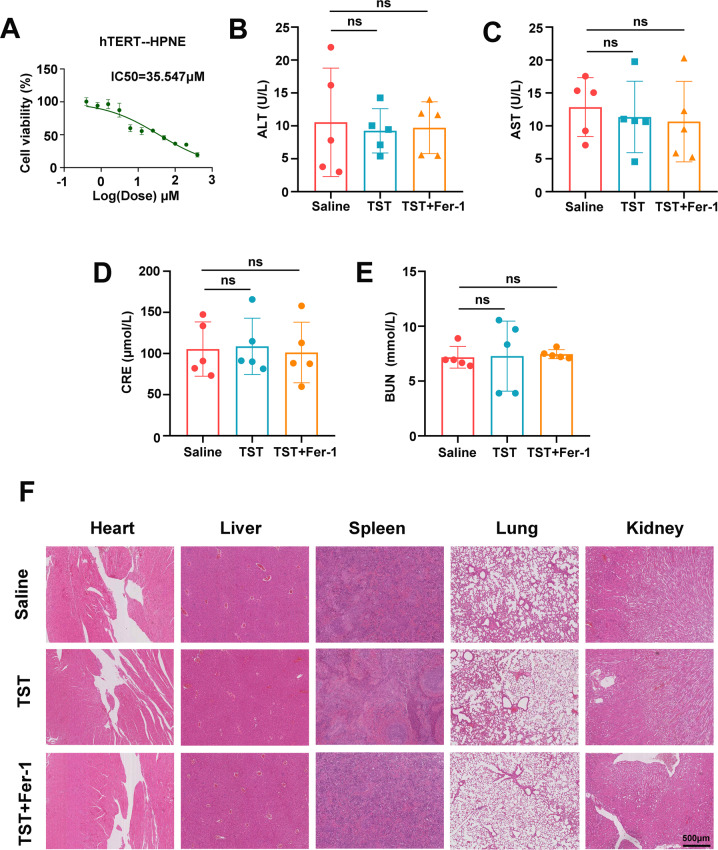
The potential biosafety value of thiostrepton on tumour treatment. **A** hTERT-HPNE cells were treated with TST (0–250 μM) for 48 h, and cell viability was determined using the CCK-8 assay. **B** and **C** Serum were collected, and the levels of ALT and AST, which are markers of liver function, in mice from different treatment groups were determined. **D** and **E** After different treatments, the levels of CRE and BUN, which are markers of renal function, were measured in mice. **F** HE staining shows the morphology of mouse heart, liver, spleen, lung and kidney from animals receiving different treatments (ns: not significant).

Previous studies suggest that TST, as a special antibiotic, has more biological value. We are looking forward to seeing if there is good safety in terms of tumour inhibition. We calculated the IC_50_ for TST in the normal pancreatic ductal epithelial cell line HPNE to examine the potential toxicity, and the results showed that the IC_50_ value was 35.574 μM (Fig. 6A), which was much higher than that in the three pancreatic cancer cell lines. Next, serum from the drug-treated mice was used to measure liver and kidney function and compared with the control group, ALT and AST levels were not significantly altered in the drug intervention group (Fig. 6B and C). The CRE and BUN results also confirmed the lack of obvious renal dysfunction in the drug-treated mice (Fig. 6D, E). Finally, we removed the heart, liver, spleen, lung and kidney tissues from the mice, fixed them and performed hematoxylin–eosin (H&E) staining. TST treatment did not cause apparent systemic toxicity, as assessed by the microscopic analysis of organ tissues (Fig. 6F). Altogether, TST is well tolerated and has a degree of biosafety in mice.

## Discussion

Pancreatic cancer is a highly aggressive tumour with a 5-year survival rate of <10% [17]. The incidence of pancreatic cancer has increased annually, but treatment strategies have changed little over the past few years. Furthermore, the recommended chemotherapy regimens are gemcitabine, FOLFIRINOX, and albumin-bound paclitaxel. However, due to the rapid and common development of chemoresistance, the effect of chemotherapy is still poor. Although many drugs have been reported to sensitize pancreatic cancer to chemotherapy in vitro, their clinical application is still years from being realized [18]. Hence, studies exploring the mechanisms of chemoresistance and the development of new adjuvant drugs to efficiently kill pancreatic cancer cells are imperative.

Ferroptosis is a nonapoptotic form of regulated cell death characterized by the accumulation of lethal levels of lipid peroxides. Emerging evidence shows that ferroptosis can be harnessed and then applied in clinical treatment strategies [19]. Furthermore, a number of drugs induce ferroptosis in PDAC cells by targeting regulatory mechanisms, such as the antimalarial drug artesunate and the antiviral drug zalcitabine. In addition, gemcitabine combined with a ferroptosis inducer has been reported to enhance the efficacy of chemotherapy. An important mechanism of chemotherapy sensitization is to reduce the expression of GPX4 in pancreatic cancer cells [20]. Therefore, inhibiting GPX4 activity or inducing GPX4 degradation is of paramount importance [21]. In addition, other key molecules in the ferroptosis pathway, such as the intracellular levels of iron, SLC7A11, GSH, ROS, HSPA5, H_2_O_2_, can be used to target ferroptosis for therapy [22].

Thiostrepton was first isolated in 1954 from *Streptomyces azureus* and used as veterinary medicine to treat bacterial infections [23]. Over the past few decades, the bioactivities of TST and its mechanisms of action have been largely investigated. In 2006, researchers found that TST downregulates the protein expression and transcriptional activity of the oncogenic transcription factor FOXM1 in cancer cells [24]. Since then, an increasing number of studies on the ability of TST to attenuate tumour progression have been reported. In addition, TST has also been reported to sensitize tumour cells to chemotherapy drugs, such as 5-FU and oxaliplatin, and increase their efficacy [25]. Most of these antitumor effects are mediated by directly targeting FOXM1, but some are not. Kroemer et al. revealed that TST promotes autophagy in cancer cells through a pathway relying on the activation of transcription factor EB (TFEB) and transcription factor E3 (TFE3) [26]. Another study suggested that TST rapidly induces oxidative stress followed by the inactivation of chymotrypsin-like proteasomal activity to kill melanoma cells [27]. Moreover, in high-grade serous ovarian cancer, PAX8 is targeted by micelle-encapsulated TST, resulting in limited tumour growth [14]. The inhibitory effect of TST on tumour growth has been well reported in previous studies, however, the role of TST in pancreatic cancer remains unclear.

In the present study, we aimed to study the therapeutic effect of TST on pancreatic cancer in vitro and in vivo and its underlying mechanisms. In vitro, we revealed that TST reduced pancreatic cancer cell viability and clonogenesis. Next, we began to explore the mechanism by which TST kills pancreatic cancer cells. Ferroptosis is characterized morphologically by the presence of smaller than normal mitochondria with condensed mitochondrial membrane densities, reduction or vanishing of mitochondria crista, and outer mitochondrial membrane rupture [8]. Using transmission electron microscopy, we found that the morphology of mitochondria in the treated pancreatic cancer cells was similar to the morphology of mitochondria in cells undergoing ferroptosis. Additionally, during the occurrence of ferroptosis, excess iron produces reactive oxygen species (ROS) through an iron-dependent Fenton reaction [28]. Here we also confirmed that TST could induce this phenomenon. Concurrently, similar to treatment with the ferroptosis inducer RSL3, TST treatment caused cellular ROS accumulation, MDA overexpression, and GSH-PX depletion; moreover, these changes were reversed by treatment with the ferroptosis inhibitor Fer-1, the inhibitory effect of Fer-1 was also consistent with other reports [29]. Based on the results from the aforementioned experiments, we proposed that ferroptosis is partially involved in pancreatic cancer cell death induced by TST. Previous studies have fully reported the inhibitory effect of TST on tumour cells [10, 26, 30], and we had confirmed this. But the difference is that we also found that TST could accomplish such an inhibitory effect by means of ferroptosis.

Next, we set out to explore the molecular mechanism by which TST induces ferroptosis. At first, we found that TST may not regulate SLC7A11 protein expression, so we considered that TST does not regulate glutamate transport by SLC7A11/xCT/system [5]. Besides, as an important regulator, GPX4 prevents ferroptosis by converting lipid hydroperoxides into nontoxic lipid alcohols [4]. Interestingly, we found TST induced ferroptosis by reducing the GPX4 expression, consistent with previous studies [31–33]. Therefore, we continued to explore how TST affects GPX4. Activation of STAT3 has been reported to modulate GPX4 expression, but the underlying mechanism is unclear. Our study confirmed that STAT3 could be bound to the GPX4 promoter region and promoted transcription, and TST blocked GPX4 expression by regulating STAT3 and p-STAT3 expression, which has not been confirmed compared with other previous studies [16, 34, 35]. Finally, in vivo experiments suggested that TST delayed the growth of subcutaneously transplanted tumours and has good biosafety. Drug safety is considered to be a key aspect of clinical medication, so our study may lay a theoretical foundation for a further clinical study.

As mentioned above, we did not study whether the regulation of FOXM1 by TST plays a role in ferroptosis but used FOXM1 expression as an indicator of the effect of TST. Therefore, in this experiment, we were unable to determine whether the regulation of STAT3 and p-STAT3 by TST is mediated by FOXM1. In addition, according to previous reports, TST targets rRNA in ribosomes and proteasomes, ultimately affecting protein translation and degradation [36, 37]. Therefore, the specific mechanism of TST-regulating STAT3 and p-STAT3 needs to be confirmed by further experiments. In addition, we only confirmed that TST could increase the intracellular iron levels, but the intrinsic mechanism also required further study to explore.

**Fig. 7:**
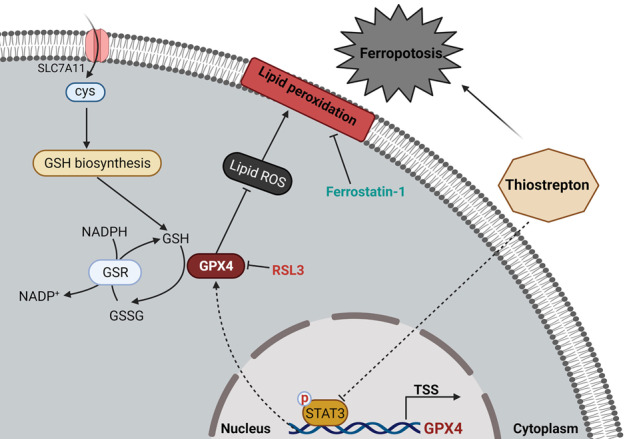
Schematic diagram of the proposed mechanism by which thiostrepton modulates ferroptosis via the STAT3/GPX4 signalling pathway. In general, in pancreatic cancer cells, TST does not regulate the function of SLC711A but instead alters the expression of STAT3. Activated STAT3 binds to the GPX4 promoter region to promote its transcription. Therefore, GPX4 downregulation by TST subsequently promotes the production of lipid ROS, leading to lipid peroxidation that ultimately induces ferroptosis.

In the present study, we showed that TST was a powerful anti-pancreatic cancer drug. It also induced ferroptosis in pancreatic cancer cells by inhibiting the STAT3/GPX4 signalling pathway (Fig. 7). This compound may represent a promising new approach to pancreatic cancer treatment. However, the specific mechanism must be elucidated and further clinical trials are needed.

## Supplementary information

Supplementary tables and figure legends

Supplementary Figure s1

Supplementary Figure s2

Original images of western blot

checklist
