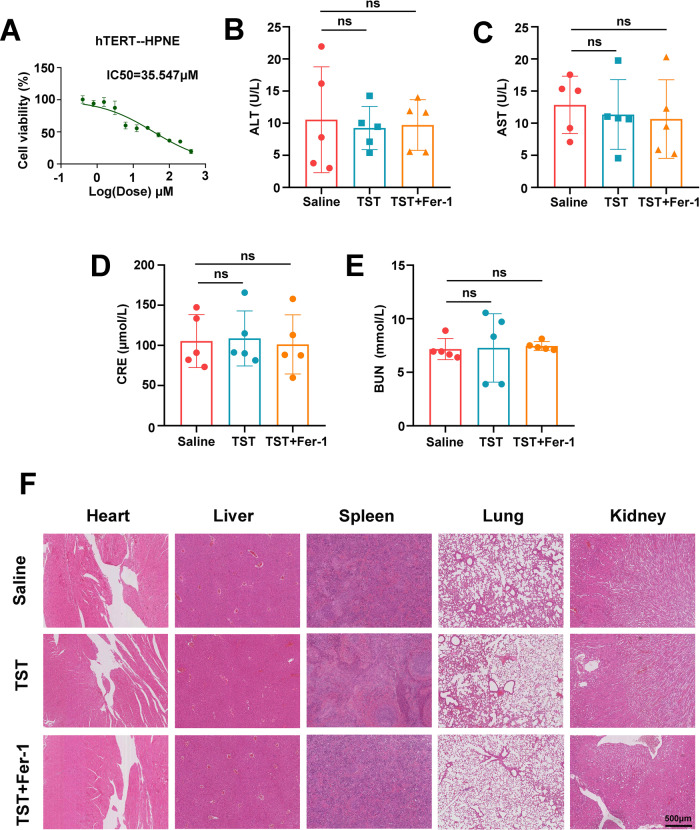# Correction: Thiostrepton induces ferroptosis in pancreatic cancer cells through STAT3/GPX4 signalling

**DOI:** 10.1038/s41419-022-05236-3

**Published:** 2022-09-14

**Authors:** Weifan Zhang, Mengyuan Gong, Wunai Zhang, Jiantao Mo, Simei Zhang, Zeen Zhu, Xueni Wang, Bo Zhang, Weikun Qian, Zheng Wu, Qingyong Ma, Zheng Wang

**Affiliations:** 1 Department of Hepatobiliary Surgery, The First Affiliated Hospital of Xi’an Jiaotong University, Xi’an, Shaanxi 710061 China; 2 Pancreas Center, Xi’an Jiaotong University, Xi’an, Shaanxi 710061 China

**Keywords:** Cancer prevention, Cancer therapy

Correction to: *Cell Death and Disease* 10.1038/s41419-022-05082-3, published online 20 July 2022

The original version of this article unfortunately contained an error in Fig. 6F. The authors apologize for the error. The correct figure can be found below. The original article has been corrected.