# Supplementary material for: Thiostrepton induces ferroptosis in pancreatic cancer cells through STAT3/GPX4 signalling
Source: Cell Death Dis. 2022 Jul 20;13(7):630. doi: 10.1038/s41419-022-05082-3 (PMC9300693; doi:10.1038/s41419-022-05082-3)
Supplement: Supplementary file 1 — Supplementary tables and figure legends [file 41419_2022_5082_MOESM1_ESM.docx]

**Supplementary figure legends**

**Figure. S1.** [Representative](javascript:;) imaging of intracellular iron levels was conducted in control and TST-treated pancreatic cancer cells.

**Figure. S2. ­­**Representative IF images showing the expression of p-STAT3. The second antibody was labelled with FITC (green), and the cell nucleus were stained with DAPI (blue).

**Supplemental Table 1.** The GPX4 primers used in chip assay

| **Primers** | **Forward (5’-3’)** | **Reverse (5’-3’)** |
| --- | --- | --- |
| *GPX4-P1* | CTGGGCGTGGTAGCACAT | GCTCCATCGCAGCTCACT |
| *GPX4-P2* | TTATACAAGCGGCTCAGCAA | TGGGTTTGCTTCTCATCACA |
| *GPX4-P3* | CCTTTGGCTCACGCATGT | GGGTCTCGCTATGTTGCCTA |
| *GPX4-P4* | GAATGGTGGATGAGCCTGTT | GTGCAGTGGCACGATCAG |
