# Supplementary figures and images for: Thiostrepton induces ferroptosis in pancreatic cancer cells through STAT3/GPX4 signalling
Source: Cell Death Dis. 2022 Jul 20;13(7):630. doi: 10.1038/s41419-022-05082-3 (PMC9300693; doi:10.1038/s41419-022-05082-3)

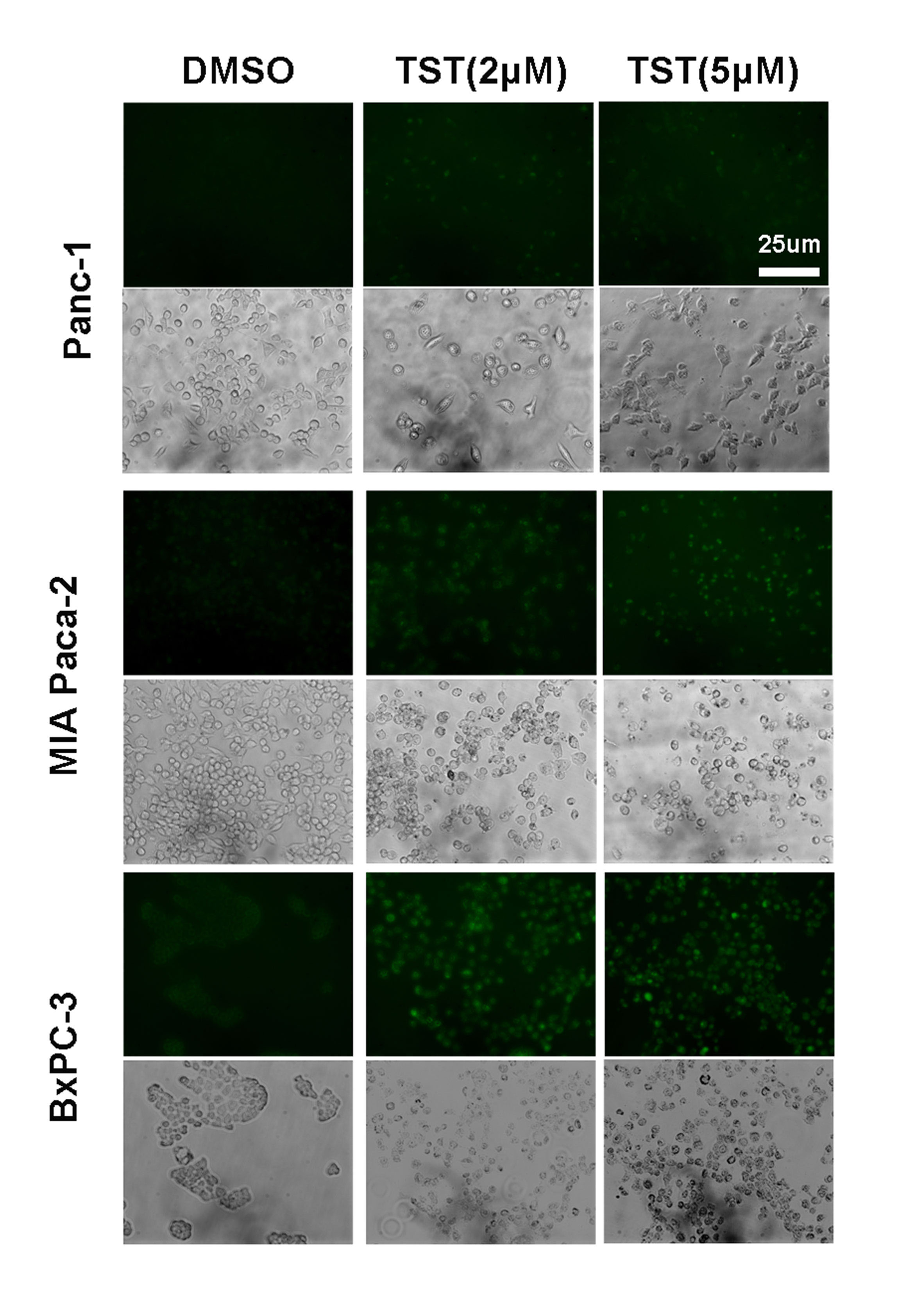

Supplement: Supplementary file 2 — Supplementary Figure s1 [file 41419_2022_5082_MOESM2_ESM.jpg]

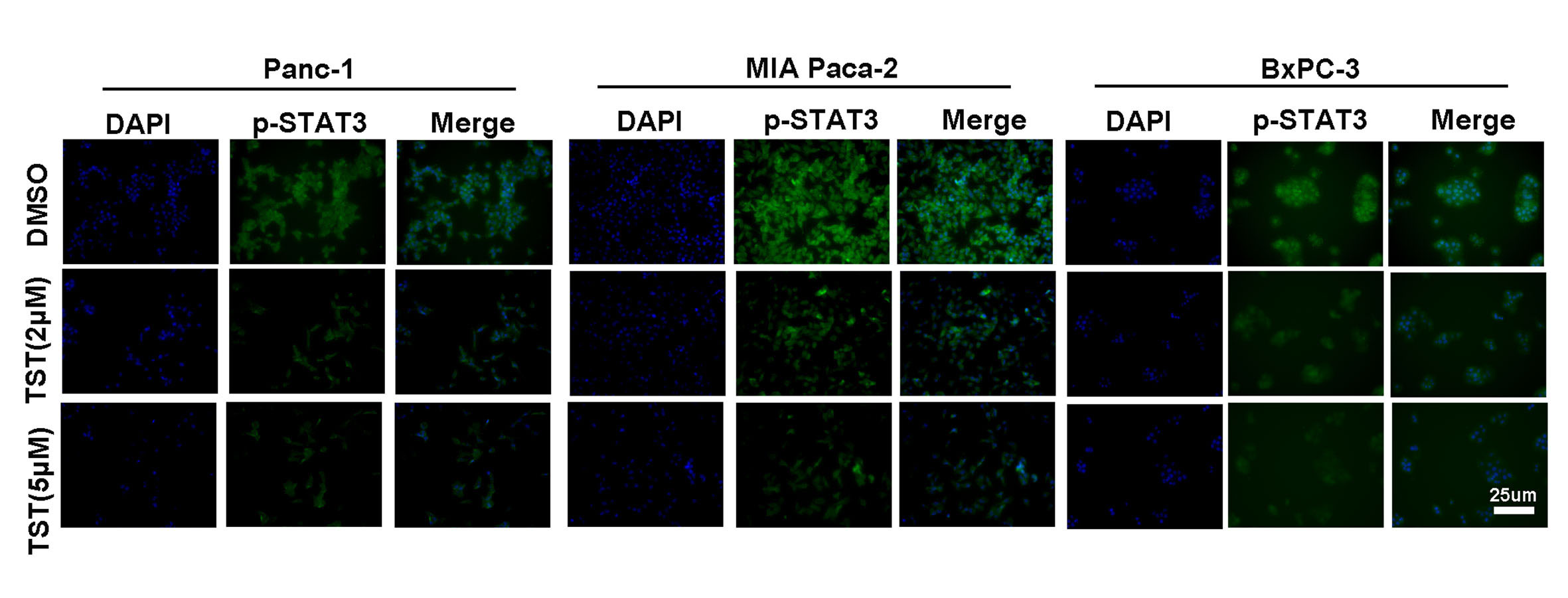

Supplement: Supplementary file 3 — Supplementary Figure s2 [file 41419_2022_5082_MOESM3_ESM.jpg]
