# Supplementary material for: Thiostrepton induces ferroptosis in pancreatic cancer cells through STAT3/GPX4 signalling
Source: Cell Death Dis. 2022 Jul 20;13(7):630. doi: 10.1038/s41419-022-05082-3 (PMC9300693; doi:10.1038/s41419-022-05082-3)
Supplement: Supplementary file 4 — Original images of western blot [file 41419_2022_5082_MOESM4_ESM.pdf]

Fig 1C

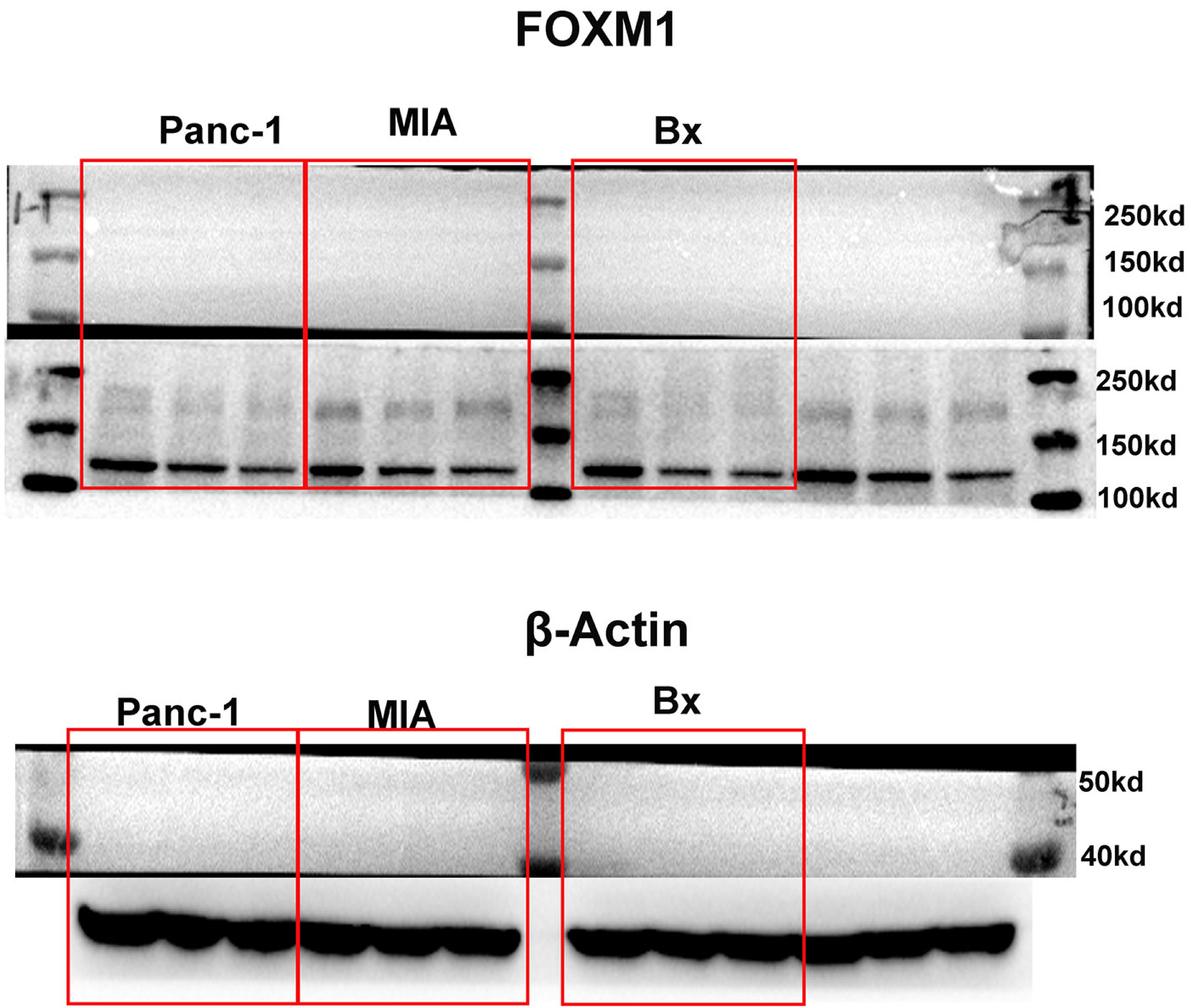
FOXM1
Panc-1
MIA
Bx
250kd
150kd
100kd
250kd
150kd
100kd
β-Actin
Panc-1
MIA
Bx
50kd
40kd

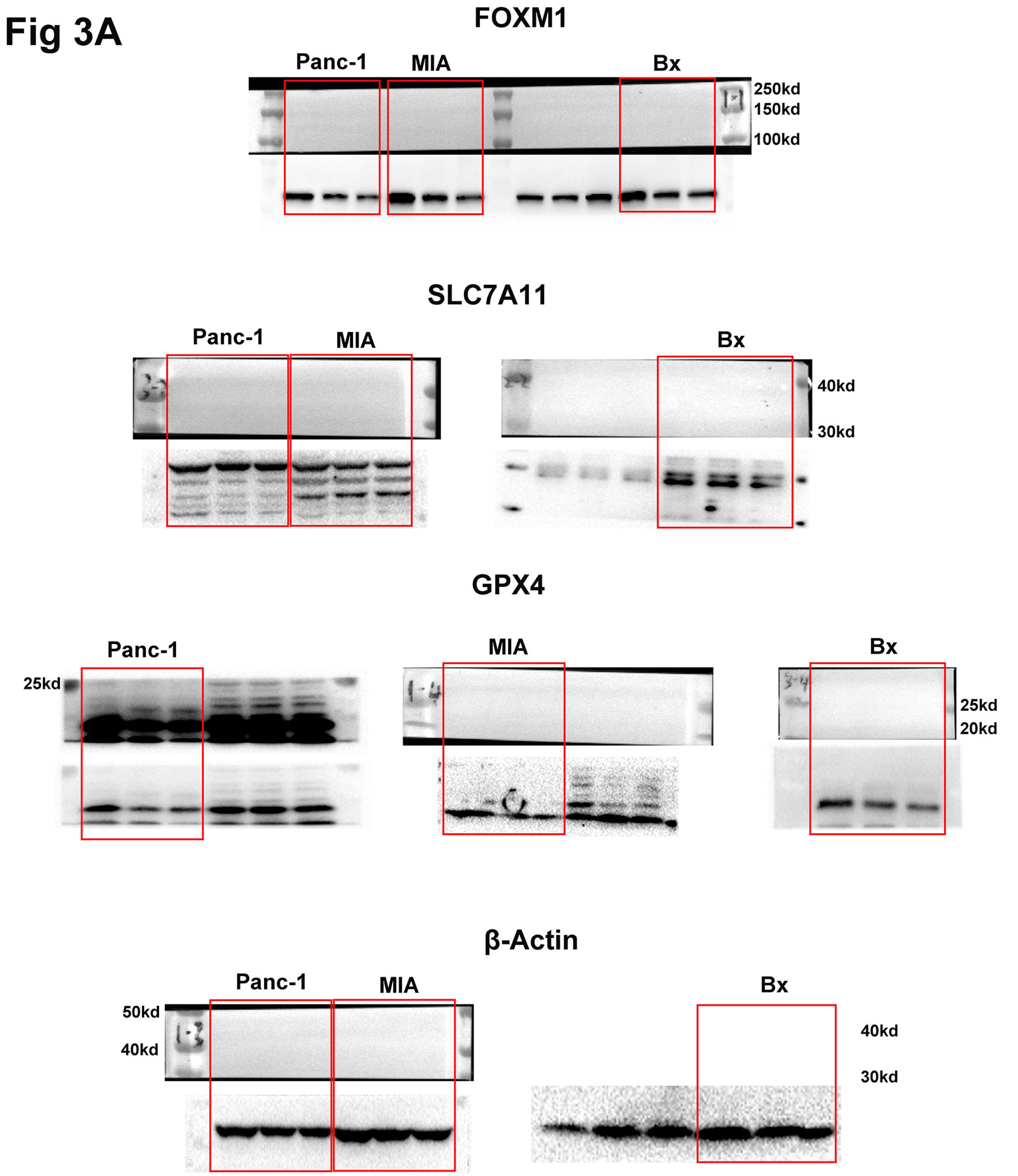
Fig 3A
FOXM1
Panc-1
MIA
Bx
250kd
150kd
100kd
SLC7A11
Panc-1
MIA
Bx
40kd
30kd
GPX4
Panc-1
25kd
MIA
Bx
25kd
20kd
β-Actin
Panc-1
MIA
50kd
40kd
Bx
40kd
30kd

Fig 3C

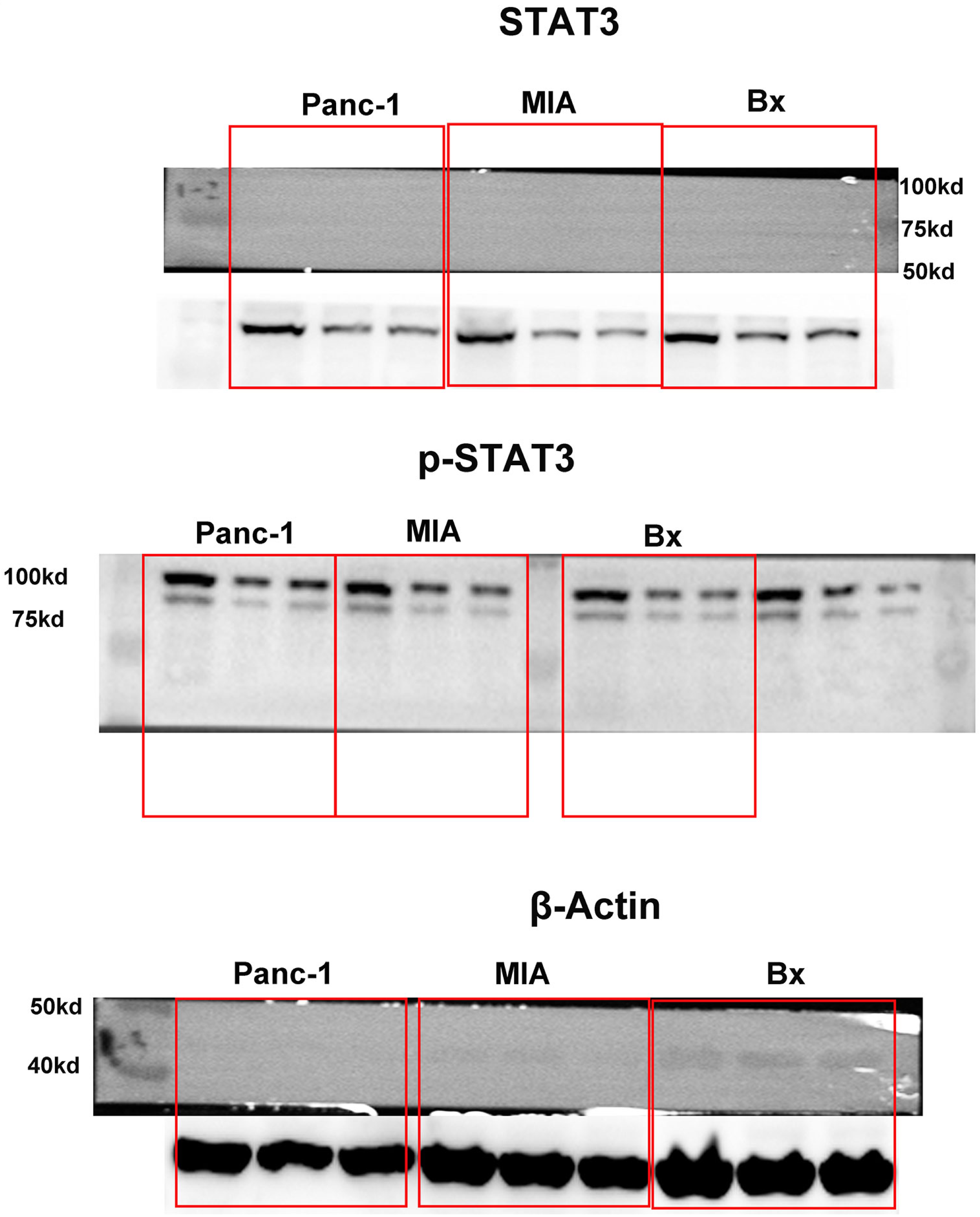
STAT3
Panc-1
MIA
Bx
100kd
75kd
50kd
p-STAT3
Panc-1
MIA
Bx
100kd
75kd
β-Actin
Panc-1
MIA
Bx
50kd
40kd

Fig 3E

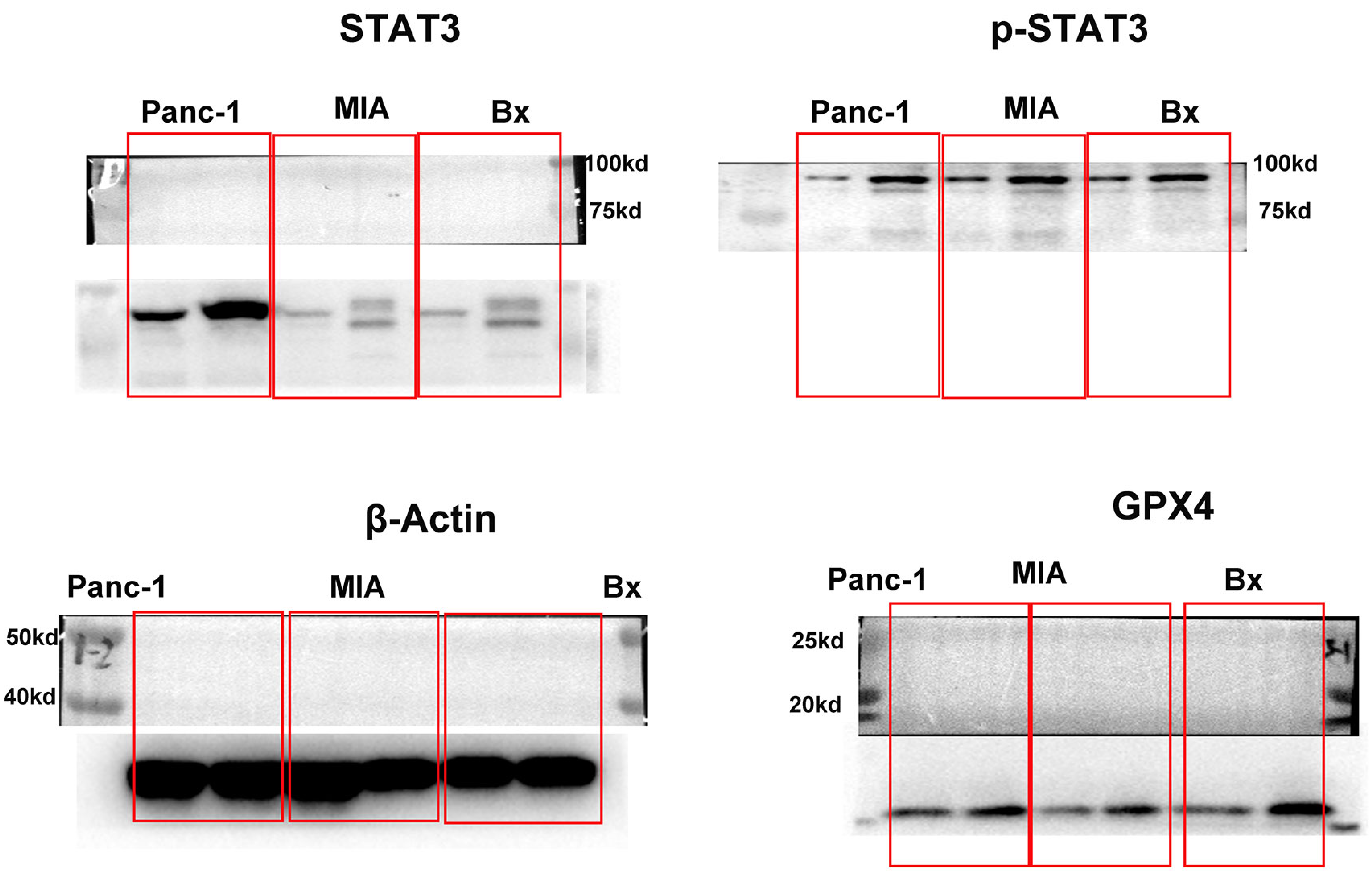

STAT3
Panc-1
MIA
Bx
100kd
75kd
p-STAT3
Panc-1
MIA
Bx
100kd
75kd
β-Actin
Panc-1
MIA
Bx
50kd
40kd
GPX4
Panc-1
MIA
Bx
25kd
20kd

Fig 4B

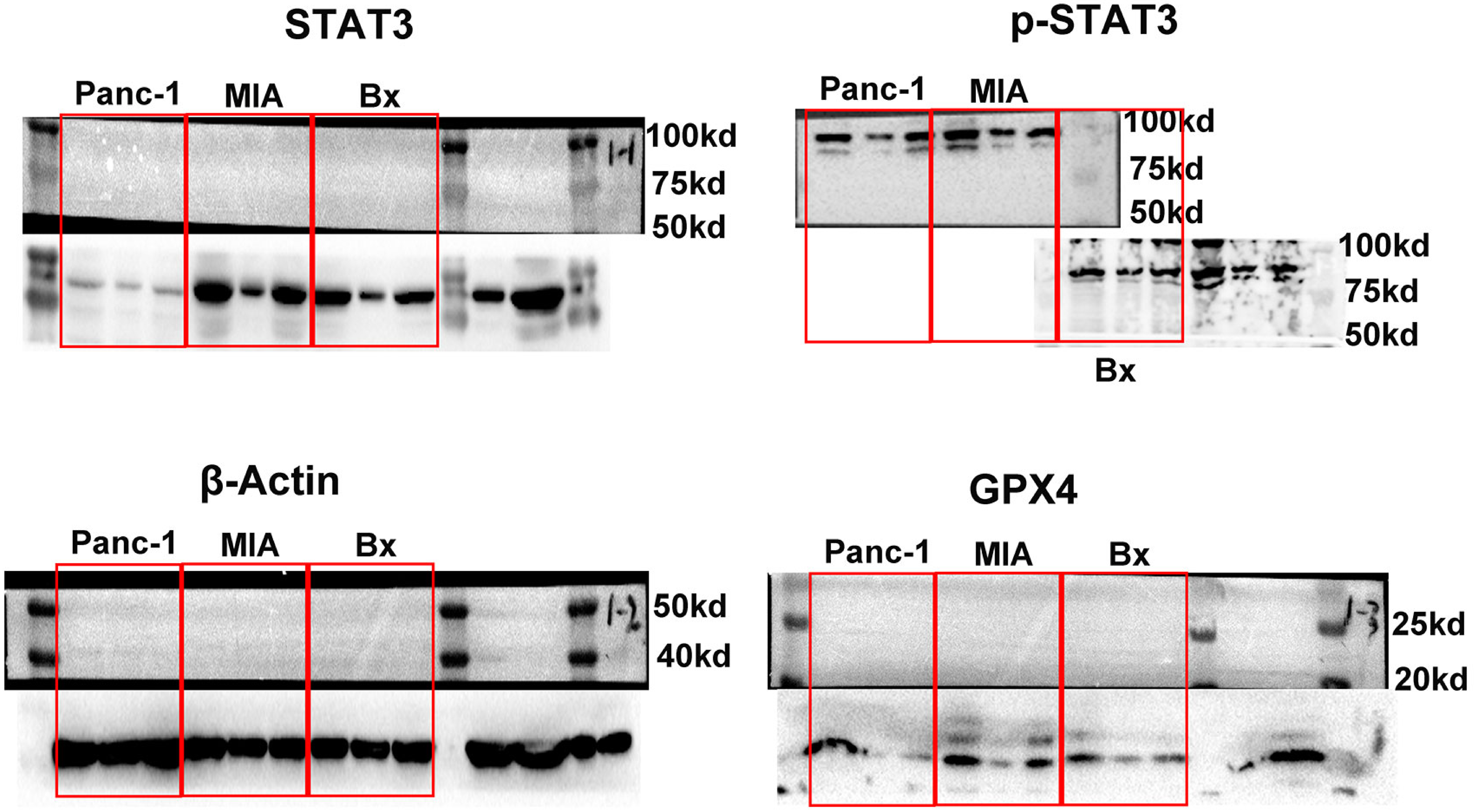
STAT3
Panc-1
MIA
Bx
100kd
75kd
50kd
1-1
p-STAT3
Panc-1
MIA
100kd
75kd
50kd
100kd
75kd
50kd
Bx
β-Actin
Panc-1
MIA
Bx
50kd
40kd
1-2
GPX4
Panc-1
MIA
Bx
25kd
20kd
1-3

Fig 5E

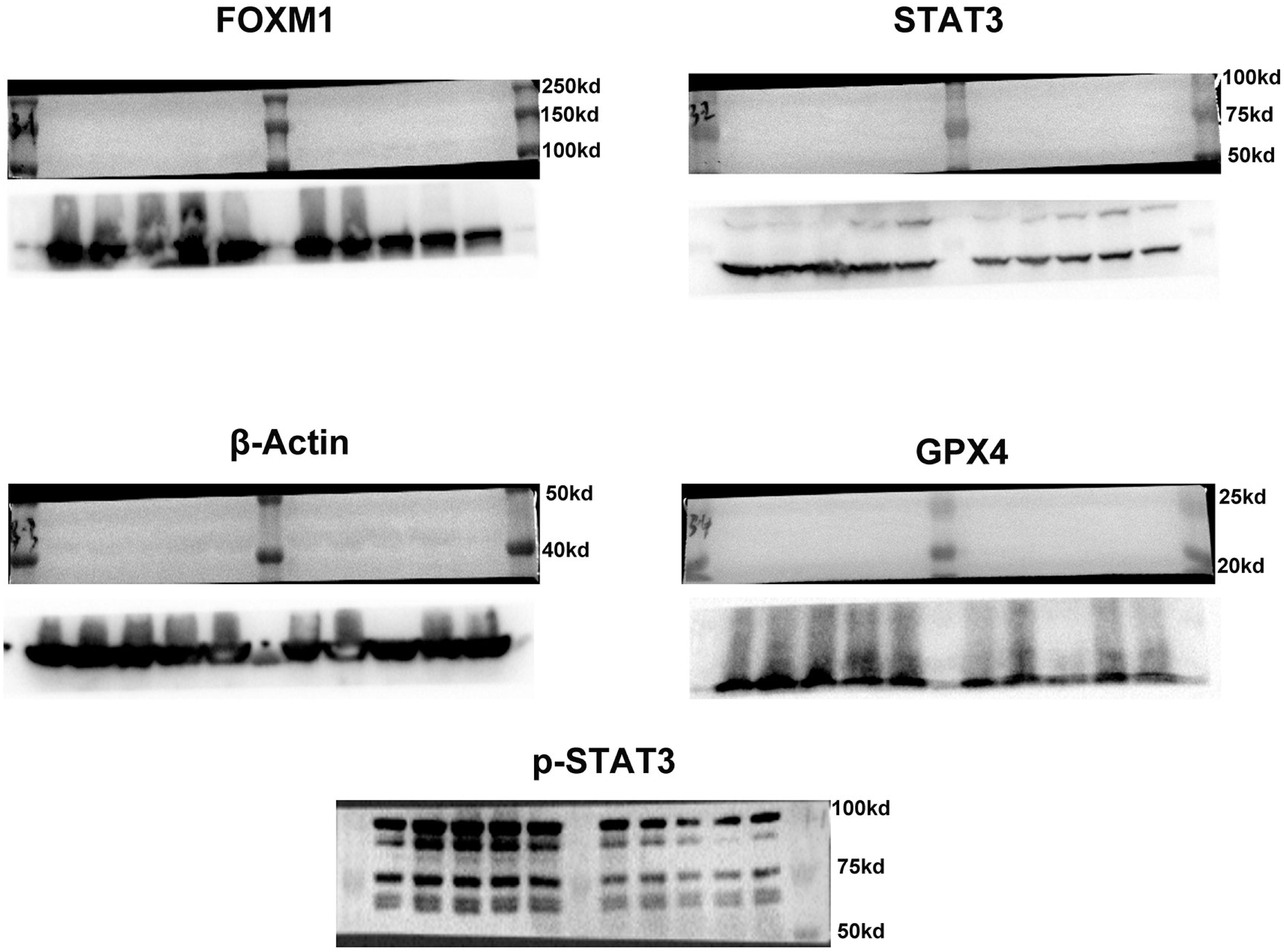
FOXM1
250kd
150kd
100kd
STAT3
100kd
75kd
50kd
β-Actin
50kd
40kd
GPX4
25kd
20kd
p-STAT3
100kd
75kd
50kd
